# Factors that Influence The Occurrence of Multiple Pregnancies after Intracytoplasmic Injection Cycles with Two or Three Fresh Embryo Transfers 

**DOI:** 10.22074/ijfs.2017.4718

**Published:** 2017-09-03

**Authors:** Mahbubeh Abdollahi, Reza Omani Samani, Mandana Hemat, Arezoo Arabipoor, Fatemeh Shabani, Farzad Eskandari, Masoud Salehi

**Affiliations:** 1 Department of Biostatistics, Faculty of Medical Sciences, Tarbiat Modares University, Tehran, Iran; 2 Department of Epidemiology and Reproductive Health, Reproductive Epidemiology Research Center, Royan Institute for Reproductive Biomedicine, ACECR, Tehran, Iran; 3 Department of Endocrinology and Female Infertility, Reproductive Biomedicine Research Center, Royan Institute for Reproductive Biomedicine, ACECR, Tehran, Iran; 4 Department of Mathematical Statistics, Faculty of Economics, Allameh Tabatabai University, Tehran, Iran; 5 Health Management and Economics Research Center, Department of Statistics and Mathematics, School of Health Management and Information Sciences, Iran University of Medical Sciences, Tehran, Iran

**Keywords:** Multiple Pregnancy, Intracytoplasmic Injection Cycles, Risk Factors, Logistic Regression

## Abstract

**Background:**

Multiple pregnancies are an important complication of assisted reproductive technology (ART). The present study aims to indentify the risk factors for multiple pregnancies independent of the number of transferred embryos.

**Materials and Methods:**

This retrospective study reviewed the medical records of patients who underwent intracytoplasmic sperm injection (ICSI) cycles in Royan Institute between October 2011 and January 2012. We entered 12 factors that affected the number of gestational sacs into the poisson regression (PR) model. Factors were obtained from two study populations-cycles with double embryo transfer (DET) and cycles that transferred three embryos (TET). We sought to determine the factors that influenced the number of gestational sacs. These factors were entered into multivariable logistic regression (MLR) to identify risk factors for multiple pregnancies.

**Results:**

A total of 1000 patients referred to Royan Institute for ART during the study period. We included 606 eligible patients in this study. PR analysis demonstrated that the quality of transferred embryos and woman’s age had a significant effect on the number of observed sacs in patients who underwent ICSI with DET. There was no significant predictive variable for multiple pregnancies according to MLR analysis. Our findings demonstrated that both regression models (PR and MLR) had the same outputs. A significant relation existed between age and fertilization rate with multiple pregnancies in patients who underwent ICSI with TET.

**Conclusion:**

Single embryo transfer (SET) should be considered with the remaining embryos cryopreserved to prevent multiple pregnancies in women younger than 35 years of age who undergo ICSI cycles with high fertilization rates and good or excellent quality embryos. However, further prospective studies are necessary to evaluate whether SET in women with these risk factors can significantly decrease multiple pregnancies and improve cycle outcomes.

## Introduction

At present, the use of assisted reproductive technology (ART) is expanding worldwide. A related challenge after ovarian hyperstimulation is multiple gestation and the health of children born by ART. Recently, the rate of multiple pregnancies has dramatically increased due to the widespread use of ART (1). Multiple pregnancies are associated with increased risk of maternal and fetal complications (2). The ideal of infertility therapy is to achieve one healthy baby at a time (3). Despite the attempts to limit the incidence of multiple pregnancies after ART by elective single embryo transfer (SET), the average *in vitro* fertilization (IVF) treatment includes transfer of two, three or sometimes more embryos into the uterus. SET, as a clinical practice, has not been executed in some countries.

Previous studies evaluated embryo and cyclespecific parameters associated with twin pregnancies after double embryo transfer (DET) (4-8). Niu et al. (4) found that four factors-the first treatment cycle, good ovarian response, higher number of top-quality embryos, and development stage score of the second-best embryo transferred had an independent association with twin pregnancies after DET. Xu et al. (5) reported that women’s age and the number of highquality embryos transferred were risk factors associated with twin pregnancies after IVF with DET. Groeneveld et al. (6) demonstrated that the height of the women and the number of oocytes retrieved were associated with an increased risk of twins after DET.

In a recent study, Kim et al. (8) concluded that younger age, higher body weight, and better quality of transferred embryos showed an association with increased chance for twin pregnancies after DET at cleavage stage. Identification of risk factors for multiple pregnancies can enable medical personnel to provide counseling and information to couples at high risk for multiple pregnancies and suggest SET in their infertility treatment program. The aim of present study is to evaluate the factors that affect the occurrence of multiple pregnancies after transfer of two or three embryos in intracytoplasmic sperm injection (ICSI) by two different regression analyses (poisson and Logistic).

## Materials and Methods

This retrospective cohort study reviewed the records of patients referred to Royan Institute between October 2011 and January 2012. The Institutional Scientific Board of Royan Institute approved this study. Admitted patients gave written consent that stated which the treatment information would be used for scientific purposes without mentioning names or personal details.

The data related to infertile couples who underwent ICSI cycles and included: demographic information, medical records, and cycle characteristics. Excluded from the study were: women older than 40 years, uterine factor infertility (myoma, polyps, and congenital malformations), recurrent miscarriages, and stages II or III endometriosis. Cycles with donor oocytes or embryos, preimplantation genetics diagnosis, and blastocyst embryo transfer were also excluded. In this study we reviewed all ICSI cycles that had complete data. Embryo quality was recorded based on the fragmentation degree and regularity of blastomeres on day 3 after fertilization, as follows (9): excellent: 6-8 equal sized blastomeres with ≤10% frag mentation; good: 6-8 equal or unequal sized blastomeres with 10-20% fragmentation; and fair: uneven sized and few blastomeres with >20% fragmentation. We calculated the fertilization rate according to the number of fertilized oocytes per number of microinjected MII oocytes. Multiple pregnancies were considered as the observation of more than one gestational sac with heart beats by vaginal ultrasound evaluation six weeks after embryo transfer.

### Statistical analysis

The findings were described as mean ± SD for quantitative variables and number (%) for qualitative variables. We compared the study population characteristics according to the number of embryos transferred, either DET or triple embryo transfer (TET) according to the student’s t test and chi-square test for quantitative and qualitative variables, respectively. To investigate the relationship between factors and gestational sac, we used the statistical software STATA 11 program and the logistic and poisson regression (PR) models. On the basis of previous studies (5, 8, 10, 11), the possible variables that affected the number of gestational sacs included: women’s age, body mass index (BMI), infertility type, infertility cause and duration, acquired uterine anomaly, endometrial thickness on the day of the human chorionic gonadotropin (hCG) injection, type of stimulation protocol, present cycle type, ovarian response type, total number of gonadotropin ampules, total number of retrieved oocytes, number of MII oocytes, fertilization rate, and quality of transferred embryos were listed in the PR model for both study populations (DET and TET). Multiple logistic regression (LR) model was used to determine significant variables related to multiple pregnancies. We considered P<0.05 to be statistically significant.

## Results

A total of 1000 patients referred to Royan Institute for ART during the study period. We included 606 patients in this study. There were 336 patients with DET and 270 patients with TET. Table 1 lists the demographic and medical characteristics of patients according to group (DET or TET). The women’s mean age, duration of infertility, and number of previous ART cycles in the TET group were greater than those in the DET group (P<0.001). The two groups showed no significant differences in terms of BMI, infertility type, endometrial thickness, and quality of transferred embryos. The DET group had a higher mean number of retrieved and MII oocytes. Despite a higher fertilization rate in the TET group, we observed clinical pregnancy rate between the two groups (P=0.1).

The distribution of the frequency of the numberof sacs in the DET and TET groups were shown in Table 2. The results showed that the mean of the observed gestational sac in the TET group (0.64 ± 0.88) was greater than the DET group (0.48 ± 0.72, P=0.01). We calculated incidence rate ratios (IRRs) for explanatory variables by the PR models to identify the related variables to the number of observed gestational sacs after the ICSI cycles. If an IRR is greater than 1, the incidence rate (IR, sac no.) increases as x (explanatory variable) increases. If an IRR is less than 1, the IR decreases as x increases.

**Table 1:** Study population characteristics in patients with double embryo transfer (DET) and three embryo transfer (TET)


Variable		DET n=336	TET n=270	P value

Women’s age (Y)		29.3 ± 5.2	32.2 ± 4.2	<0.0001^*^
Body mass index (BMI)		25.2 ± 3.7	25.7 ± 3.8	0.1
Diagnosis infertility				0.09
	Female	116 (34.5)	112 (58.5)	
	Male	220 (65.5)	158 (41.5)	
Duration of infertility		5.9 ± 4.2	7.5 ± 5.3	<0.0001^*^
Type of infertility				0.5
	Primary	270 (80.4)	212 (78.5)	
	Secondary	66 (19.6)	58 (44.6)	
Previous ART attempts		1.09 ± 1.2	1.6 ± 2.0	<0.0001^*^
Number of used gonadotropin ampules		26.8 ±11.1	29.1 ±11.3	0.01^*^
Endometrial thickness on hCG day		10.3 ±1.8	10.3 ± 2.0	0.6
Total number of retrieved oocytes		9.9 ± 5.1	8.6 ± 3.5	0.001^*^
Number of MII oocytes		8.4 ± 4.5	7.6 ± 3.1	0.009^*^
Quality of transferred embryos				0.1
	Fair	28 (8.4)	14 (5.2)	
	Excellent	48 (14.3)	34 (12.6)	
	Good	260 (77.3)	226 (82.2)	
Fertilization rate		0.59 ± 0.24	0.65 ± 0.22	0.002^*^
Clinical pregnancy rate		119 (35.4)	112 (44.6)	0.1


Results are presented as mean ± SD, n (%). ART; Assisted reproductive technology, hCG; Human chorionic gonadotropin, and *; Significant at the 0.05 level.

**Table 2:** Distribution of the frequency of observed gestational sacs in the double embryo transfer (DET) and triple embryo transfer (TET) groups


Number of sacs	DET n=336	TETn=270	P value
	Frequency (%)	Frequency (%)	

0	217 (64.6)	158 (58.5)	0.01^*^
1	78 (23.2)	62 (23)	
2	39 (11.6)	38 (14.1)	
3	3 (0.6)	12 (4.4)	


*; Significant at the 0.05 level.

The quality of the transferred embryos and woman's age significantly impacted the number of observed sacs in the DET group (Table 3). It means that the IR of multiple sac in excellent grade embroyos group was 9 times and meanwhile in good grade embroyos group was 6 times respect to fair grade embryos as refrence group. The results showed that the IRR for women’s age was 0.96 (95% CI: 0.93- 0.99). In other words, one year increases in female age showed an IR of the observed gestational sac that decreased 4%. The other explanatory variables did not impact the number of sacs.

In the TET group, women's age, type of infertility, and fertilization rate significantly impacted the number of sacs (Table 4). The IRR for maternal age was 0.94 (95% CI: 0.90-0.97) and IRR for the fertilization rate was 6.02 (95% CI: 2.96-12.22). The one year increase in female age showed an IR of the observed gestational sac that decreased 6%. The IR of the observed gestational sac increases 6 times when one unit increases in the fertilization rate. The results demonstrated that the IR of the observed gestational sac in patients with secondary infertility decreased 42% with respect to those with primary infertility (Table 4).

All the possible affecting variables that included women’s age, BMI, infertility type, infertility cause and duration, acquired uterine anomaly, endometrial thickness on the day of the hCG injection, type of stimulation protocol, present cycle type, ovarian response type, total number of gonadotropin ampules, total number of retrieved oocytes, number of MII oocytes, fertilization rate, and quality of transferred embryos were entered in the multiple logestic regression (MLR) model. The result showed that women's age, duration of infertility, and number of transferred embryos were the most important variables related to multiple pregnancies in this population (Table 5). Therefore, we repeated the MLR analysis in the two separate populations (DET and TET). The results showed that none of the variables in the model had a significant association with multiple pregnancies in the DET group. Multiple LR showed that in the TET group, women's age and fertilization rate were significantly related variables (Table 6). Women with increased age had a 20% reduction in the odds for multiple pregnancies. When the fertilization rate increased one unit, the odds for a multiple pregnancy increased 19.7 times.

**Table 3:** Results of poisson regression for assessing the effect of explanatory variables on number of sac in patients with double embryos transfer


Variable		Coefficient	SE	P value	IRR (95% CI)

Women age		-0.05	0.02	0.004^*^	0.96 (0.93-0.99)
Quality of transferred embryos					
	Excellent	2.20	0.73	0.003^*^	9.00 (2.16-37.50)
	Good	1.79	0.71	0.012^*^	6.01 (1.48-24.37)
	Fair	Reference	-	-	-


SE; Standard error, IRR; Incidence rate ratio; CI; Confidence interval, and * ; Significant at the 0.05 level.

**Table 4:** Results of poisson regression for assessing the effect of explanatory variables on number of sac in patients with three embryos transfer


Variable		Coefficient	SE	P value	IRR (95% CI)

Women age		-0.07	0.02	<0.001^*^	0.94 (0.90-0.97)
Type infertility					0.58 (0.37-0.90)
	Primary	Reference	-	-	
	Secondary	-0.55	0.23	0.016	
Fertilization rate		1.79	0.36	<0.001^*^	6.02 (2.96-12.22)


SE; Standard Error; IRR; Incidence rate ratio; CI; Confidence interval, and * ; Significant at the 0.05 level.

**Table 5:** Multiple logistic regression analysis by backward manner for predicting multiple pregnancy in all study population


Variable		OR	CI	P value^*^

Women age		0.93	(0.87-0.99)	0.04
Duration of infertility		1.07	(1.00-1.15)	0.03
No. of transferred embryos				
	Three embryos	1.73	(0.96-3.10)	0.06
	Two embryos	Reference		


OR; Odds ratio, CI; Confidence interval, and *; Significant at the 0.05 level.

**Table 6:** Multiple logistic regression analysis by backward manner for predicting multiple pregnancy in women with three embryos transfer in ICSI cycles


Variable	OR	CI	P value^*^

Women age	0.8	(0.79-0.99)	0.06
Fertilization rate	19.7	(2.6-100.6)	0.04


OR; Odds ratio, CI; Confidence interval, ICSI; Intracytopasmic sperm injection. and *; Significant at the 0.05 level.

## Discussion

In present study, on the basis of the PR model, we found that the quality of transferred embryos and women’s age had a significant effect on the number of observed sacs in patients who underwent ICSI cycles with DET. However, based on the multiple LR model, we found no significant predictive variable for multiple pregnancy in these patients. Previous studies reported that maternal age (5, 8), body composition (6, 8), good ovarian response, cycle number, and the number of retrieved oocytes (4) had a relationship to multiple pregnancies after DET. The current study results showed no impact by body composition, cycle number, number of retrieved oocytes, and type of ovarian response. In agreement with previous studies (5, 8), we found that the quality of transferred embryos significantly influenced the IR of sac numbers. Kaser et al. (7) stated that there were six risk factor for twin live births after cryopreserved cleavage stage DET cycles: age <35 years, resumption of mitosis, 7-8 viable cells in the non-lead embryo, transfer of a lead embryo with ≥7 cells and a total of ≥14 viable cells.

Groeneveld et al. (6), in a large nationwide Dutch cohort, demonstrated that tall stature and increased number of retrieved oocytes independently increased the chance for dizygotic twins after IVF with DET. In contrast to this study, we did not find any relationship between multiple pregnancies and BMI or number of retrieved oocytes in ICSI cycles with DET.

In agreement with Niu et al. (4), we found that excellent and good quality transferred embryos independently increased the chances of multiple implantation after ICSI with DET. They suggested that it was advisable to perform SET when patients had high risk factors for twin pregnancies that included initial IVF-ET treatment, good or high ovarian response, more number of top-quality embryos, and development stage score of the second best embryo transferred. Fauque et al. (12) concluded that not only the implantation and pregnancy rates, but also the live birth rate depended on embryo quality.

On the basis of our knowledge, the present study was the first to evaluate risk factors for multiple pregnancies in patients who underwent ICSI with TET. Our findings demonstrated that both regression models (poisson and logistic) had the same outputs. In both models, women’s age and fertilization rate showed significant relationships with multiple pregnancies in these patients. It could be taken as a measure of oocyte quality, which has been shown to be associated with increased implantation potential. A higher rate of fertilization would likely result in a higher number of MII oocytes and, consequently, a higher chance of good quality embryos would be associated with higher chances for multiple pregnancies. In agreement with previous studies we found a negative correlation between female age and multiple pregnancies in ICSI cycles with TET. Younger women had increased implantation potential and multiple pregnancies (10, 11).

In the present study, we have sought to evaluate the risk factors of multiple pregnancies after transfer of two or three embryos in ICSI cycles. If the risk factors of multiple pregnancies could be identified, we can give proper counseling to couples at risk for multiple pregnancies and suggest SET in their infertility treatment programs.

Currently an ideal target is considered in ART; a healthy baby is the success, not a positive pregnancy. The majority of twin or high order multiple pregnancies (HOMP) are not successful or associated with poor maternal and neonatal outcomes (13). De Neubourg and Gerris (14) have reported that the twin rate after IVF/ICSI dropped by at least 50% simply by transferring only one goodquality embryo in the first and second fresh IVF/ ICSI cycles in young women, without a reduction in the overall pregnancy rate. They believed that preventing ‘the second half‘ of IVF/ICSI twins constituted another, probably tougher challenge because the target group was a heterogeneous mix consisting of patients in very different clinical situations. However, they suggested expanding the SET policy to women <38 years of age until the third cycle and to cryoperservation cycles. In many European countries the SET policy is the primary prevention method used to prevent multiple pregnancies. However, in Iran, the transfer of just one embryo, even with excellent morphology and quality, is taboo because it is feared that the pregnancy rate will decline (15). Despite warnings from physicians about the risks associated with multiple pregnancies, patients that have longterm infertility problems perceive twin or multiple pregnancies as blessings from God. In some cases they are not satisfied with multifetal pregnancy reduction. However, physicians are ethically obligated to inform the patients regarding the risks of multiple pregnancies.

A limitation of the present study is its retrospective nature; therefore, we could not evaluate the effect of some possible factors reported in previous studies.

## Conclusion

We suggest the SET policy for prevention of multiple pregnancies in women younger than 35 years of age who undergo ICSI cycles with high fertilization rates and excellent or good quality embryos. The remaining embryos should be cryopreserved. However, further prospective studies are necessary to evaluate whether SET in women with these risk factors can significantly decrease the multiple pregnancy rate and improve cycle outcomes.
